# Anatomic evaluation of the triceps tendon insertion at the proximal olecranon regarding placement of fracture fixation devices

**DOI:** 10.1007/s00276-022-02921-y

**Published:** 2022-03-17

**Authors:** Sebastian Wegmann, V. Rausch, M. Hackl, T. Leschinger, M. Scaal, L. P. Müller, K. Wegmann

**Affiliations:** 1 Center for Orthopedic and Trauma Surgery, Faculty of Medicine, University of Cologne, University Hospital Cologne, Cologne, Germany; 2 Faculty of Medicine, Department of Anatomy II, University of Cologne, Cologne, Germany; 3 Department for Orthopedic and Trauma Surgery, University Hospital Cologne, University of Cologne, Kerpener Str. 62, 50937 Köln, Germany

**Keywords:** Triceps, Olecranon, Double plating, Elbow surgery, Fractures

## Abstract

**Purpose:**

Olecranon fractures, especially with a small proximal fragment, remain a surgical challenge. Soft tissue irritation and affection of the triceps muscle bear a risk of complications. In order to find an area for a soft-tissue sparing placement of implants in the treatment of olecranon fractures, we aimed to define and measure the segments of the proximal olecranon and evaluate them regarding possible plate placement.

**Methods:**

We investigated 82 elbow joints. Ethical approval was obtained from the local ethics committee, After positioning in an arm holder and a posterior approach we described the morphology of the triceps footprint, evaluated and measured the surface area of the triceps and posterior capsule and correlated the results to easily measurable anatomical landmarks.

**Results:**

We found a bipartite insertional footprint with a superficial tendinous triceps insertion of 218.2 mm^2^ (± 41.2, range 124.7–343.2), a capsular insertion of 159.3 mm^2^ (± 30.2, range 99.0–232.1) and a deep, muscular triceps insertion area of 138.1 mm^2^ (± 30.2, range 79.9–227.5). Olecranon height was 26.7 mm (± 2.3, range 20.5–32.2), and olecranon width was 25.3 mm (± 2.4, range 20.9–30.4). Average correlation between the size of the deep insertion and ulnar (*r* = 0.314) and radial length (*r* = 0.298) was obtained.

**Conclusions:**

We demonstrated the bipartite morphology of the distal triceps footprint and that the deep muscular triceps insertion area by its measured size could be a possible site for the placement of fracture fixations devices. The size correlates with ulnar and radial length.

**Supplementary Information:**

The online version contains supplementary material available at 10.1007/s00276-022-02921-y.

## Introduction

Olecranon fractures make up 10% of all fractures of the upper extremity with an incidence of 12/100,000 [9–11, 16, 17]. The injury pattern varies and ranges from simple non-displaced fractures to complex fracture dislocations. They are usually considered intra-articular injuries and, therefore, require anatomical restoration of the articular surface. Several surgical techniques are described in the treatment of these injuries such as tension band wiring, plate fixation, or intramedullary screw fixation [22, 31]. Plate osteosynthesis has shown favorable results regarding stability and functional outcome and is the preferred method in comminuted fracture patterns [11, 15, 28]. Another technical aspect when using dorsal olecranon plates is the placement on top of the triceps tendon, which might lead to compression of the tendon and kinking of the distal tendon in extension of the elbow joint which facilitates irritation of the tissues. A technique evading involvement of the triceps tendon would be attractive, yet does not seem possible as literature shows that the Olecranon is almost entirely covered by triceps tendon insertion and capsular tissue [3, 20, 33]. Previous studies have already investigated the triceps tendon insertion to the proximal olecranon [18, 32]. Furthermore, the insertion of the distal triceps tendon is particularly of interest in a rare tendinous injury, the distal triceps avulsion [29]. Surgical treatment is still an operative challenge as there are only little guidelines available, but will influence the patient’s outcome significantly [25, 27].

The aim of this study was to perform an anatomic investigation of the proximal olecranon, to define and measure the insertional footprint of the triceps as well as the capsular insertion on the dorsal side of the proximal olecranon in order to determine if an area would be large enough to accommodate osteosynthesis material and to assess the anatomic character of the distal triceps insertion in a larger sample size as done in previous studies in order to evaluate it for surgical repair of distal avulsions of the triceps tendon. Further, we planned to correlate the regional characteristics of that anatomical area with individual anatomic parameters which could be useful for surgical planning.

## Material and methods

The study was approved by the local ethics committee (19–1632). For the present anatomical study, 82 paired elbows of 41 formalin embalmed specimens (20 males, 21 females) were available. Specimens were excluded if there was evidence of osteoarthritis, previous injuries, relevant arthritic deformities or previous operative treatments to the elbow joint. The mean age of the donors was 84 years with SD of 9 years (range 62–101). For easier handling, the specimens were detached from the torso at the shoulder joint. Then the upper arm was placed over an arm holder, flexing the elbow joint at an angle of 90° degrees.

In each specimen, we used a posterior approach to gain access to the olecranon tip. A standard posterior skin incision was performed, which resulted in exposure of the subcutaneous tissue. Subsequently, the soft-tissues surrounding the olecranon were carefully removed, exposing the triceps muscle. The triceps was cut horizontally approximately 10 cm proximal to the olecranon tip and flipped dorsal. The triceps heads were separated and followed distally while freeing it from the medial and lateral intermuscular septae.

**Fig. 1:**
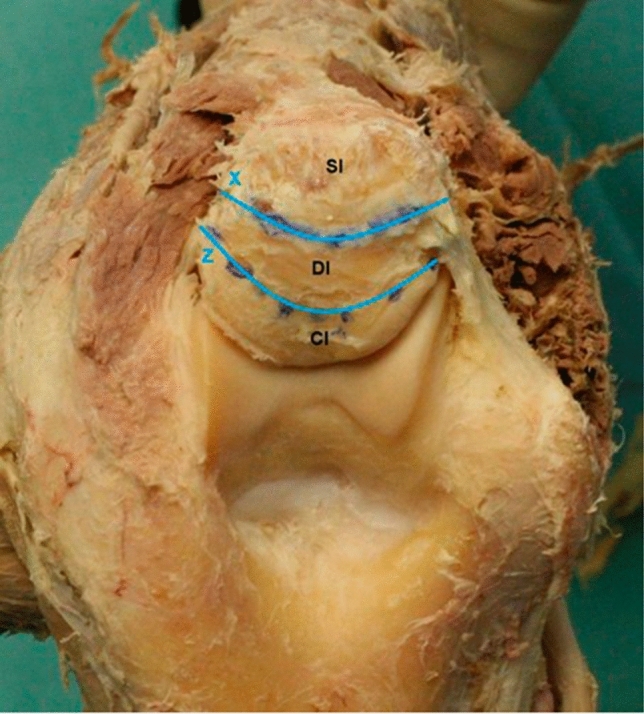
Dorsal view: After the removal of the soft tissue, line *X* was drawn with a permanent marker to limit the different triceps insertion areas (deep and superficial) visually and line *Z* was drawn to limit the distal limits of the capsular insertion (CI). Between line *X* and *Z* the limitations of the deep muscular insertion of the triceps (DI) became visible

With great care the triceps insertions were then dissected of the dorsal joint capsule, to leave the insertion of the joint capsule to the olecranon tip unharmed. Then the insertional borders of the tendon and of the joint capsule were dissected off the bony olecranon and lined out with waterproof markers. During that process we found the capsular insertion (CI) and the known tendinous part of the distal triceps tendon (SI) located superficial to a deeper located, muscular insertion (DI), which was muscular almost up to the transition to the bone. Following that, the areas were marked and the surface expanse calculated by digital imaging (Fig. 1).

Then a calibrated image was acquired perpendicular to the Olecranon tip with a millimeter-scale next to the sample. To avoid image distortion, the images were taken with a high-resolution digital camera (Canon EOS 5D) with a 50-mm fixed focal length.

Preparation and photography were repeated for all specimens in a standardized fashion. Images were then transferred into a digital image analysis software (ImageJ software, http://imagej.net).

To achieve precise and comparable measurements, each image was separately scaled using the mm-scale. This method was used before [26]. To assess the estimated error ten objects with known dimensions were measured using the same technique. We found a standard uncertainty of 1%, which we deem acceptable.

**Fig. 2:**
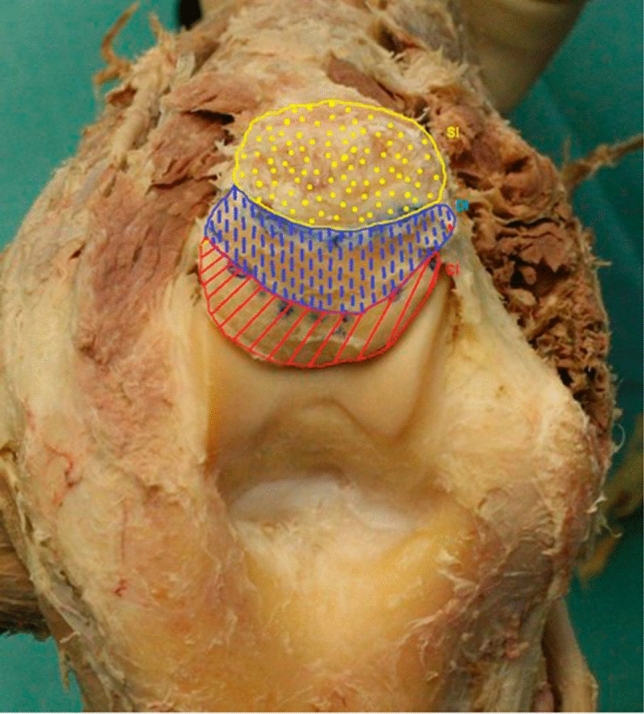
Dorsal view on the Olecranon tip. Marked and digitally measured areas: red = capsular insertion (CI), blue = deep, muscular insertion (DI), yellow = superficial, tendinous (SI)

The three areas on the digital images were lined out and the surface area was calculated with the following software: Capsular insertion (CI); deep, muscular insertion (DI); superficial, tendinous insertion (SI) (Fig. 2).

**Fig. 3:**
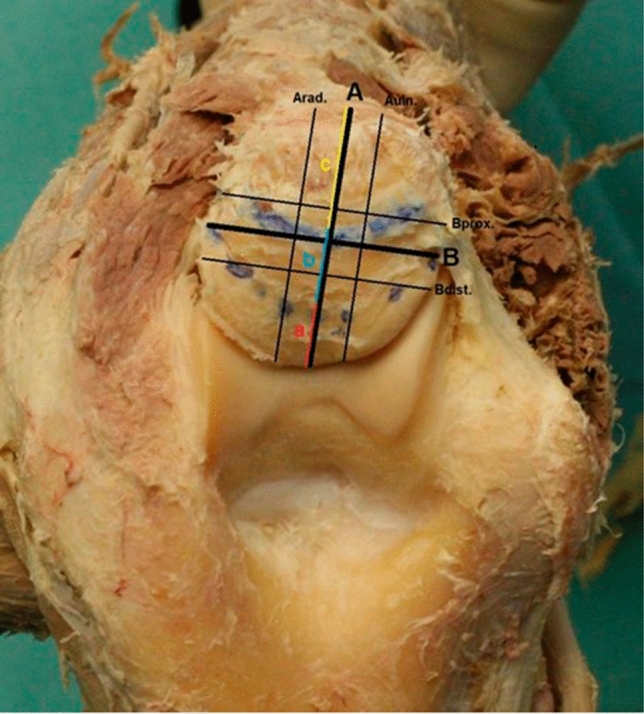
Digitally constructed and measured line A (= Olecranon height) and B (= Olecranon width), as well as distances a (= height of capsular insertion), b (= height of deep insertion of triceps) and c (= height of superficial triceps insertion). Line Arad. and Auln. as well as line Bdist. and Bprox. are 5 mm to each side of line A respectively line B

To determine the Olecranon height (OH) we digitally constructed a straight line between the tip of the olecranon and the most dorsal aspect of the triceps insertion (Line “A”). Subsequently, we constructed a perpendicular line to line “A” in order to measure the Olecranon width at the widest point, named line “B”. A second and third lines were drawn 5 mm parallel to each side of line “A” and “B” (Fig. 3). To assess the height of the previously mentioned surface areas, we measured the distance of line “A” when it intersects with the ventral and dorsal boundaries of the area. The distance between the most ventral aspect of the Olecranon and the intersection of line “A” with the insertion area of the capsule was line “a”. The distance between the intersection points of Line “A” with the deep muscular insertion area of triceps was line b and last the distance between the intersection points of line “A” with the superficial triceps insertion area was measured line “c”.

To account for differences in the specimens’ height and to correlate the results of our measurements with the individual anatomy, we measured the total length of the ulna (coronoid process to base of ulnar styloid process in cm) and the total length of the radius (radial styloid process to the articular surface of the radial head in cm) with a measuring tape.

### Statistical analysis

An a priori power analysis was performed using G*Power statistical analyzing tool to determine detectable differences in the independent variables [14]. A sample size of 35 specimens per group would provide 95% power at an alpha level of 0.05 and a Cohen´s d of 0.8.

For each of the parameters the mean, minimum, maximum, the standard deviation (SD) and the 95% confidence interval were calculated.

Validation of normal distribution of the data was done by using the Kolmogorov–Smirnov and Shapiro–Wilk test. To analyze statistical significance, the t-test for independent variables was performed, and a *p* value of < 0.05 was considered statistically significant.

To assess if there was a correlation between gender and the size of the parameters or the side of arm and the size of DI a t-test for independent samples was performed. Subsequently, Cohen’s d for effect size and the sample size for the t-test for independent samples were calculated using Microsoft Excel (Microsoft Corp., Redmond, WA, USA).

Furthermore, we calculated the Pearson correlation coefficient to analyze linear correlation between the deep triceps insertion area and the ulnar length, radial length, size of triceps insertion and size of capsular insertion in order to find coherence.

## Results

The mean area of the deep muscular insertion (DI) was 138.1 mm^2^ (SD 30.2; range 79.9–227.5). The mean superficial, tendinous insertion area (SI) was 218.2 mm^2^ (SD 41.2; range 124.7–343.2) and the mean capsular insertion (CI) was 159.3 mm^2^ (SD 30.2; range 99.0–232.1). The mean olecranon height (OH) was 26.7 mm (SD 2.3; range 20.5–32.2) and the mean olecranon width (OW) was 25.3 mm (SD 2.4; range 20.9–30.4). The results are summarized in Table 1.

**Table 1:** All measurements in the study with their mean, standard deviation and range

	Mean	SD	Min–Max
Deep insertion area (DI) in mm^2^	138.1	30.2	79.9–227.5
Superficial insertion area (SI) in mm^2^	218.2	41.2	124.7–343.2
Capsular insertion (CI) area in mm^2^	159.3	30.2	99.0–232.1
Olecranon height (OH) in mm	26.7	2.3	20.5–32.2
Olecranon width (OW) in mm	25.3	2.4	20.9–30.4
Ulnar length in cm	23.1	1.5	19.3–26.3
Radial length in cm	23.8	1.6	20.0–27.0
maximum height CI	7.4	1.5	4.3–10.8
maximum height DI	5.5	1.3	2.9–10.2
maximum height SI	12.0	1.5	7.2–16.4

For the Kolmogorov–Smirnov and Shapiro–Wilk test all results were normally distributed. The results are summarized in Table 2.

**Table 2:** *p *values for normal distribution using Kolmogorov–Smirnov test and Shapiro–Wilk test. Statistically significant results marked with*

	Kolmogorov–Smirnov	Shapiro–Wilk	
DI	0.069*	0.007	
Height DI	0.059*	0.071*	
SI	0.086*	0.053*	
CI	0.053*	0.336*	
OH	0.051*	0.815*	
OW	0.086*	0.04	
Ulnar length	0.069*	0.368*	
Radial length	0.069*	0.203*	

**Fig. 4:**
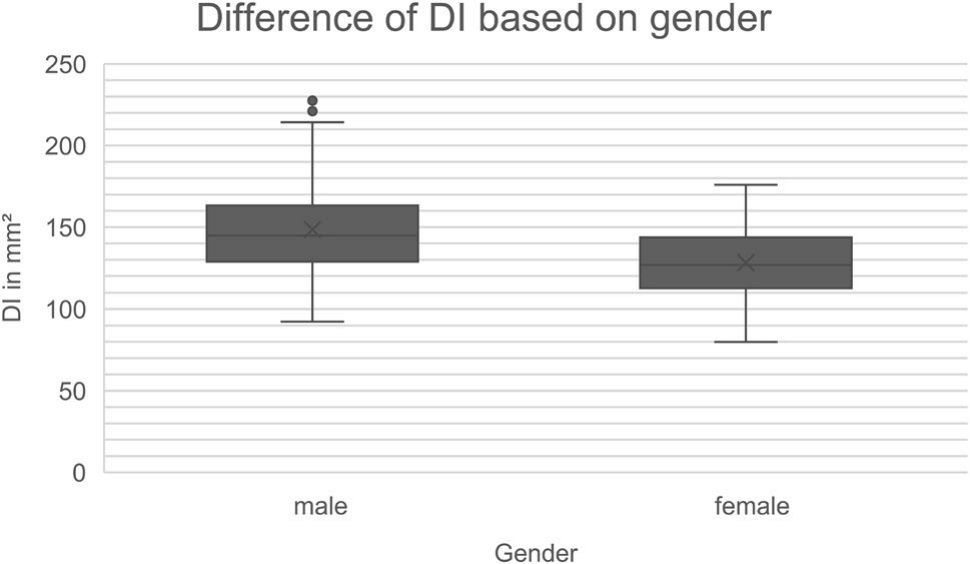
Boxplot graph visualizing the differences of the deep muscular insertion area (DI) between the male and female sex

We found the deep muscular area was 128.1 (SD 24.6) mm^2^ in female specimens while in the male cohort it was 148.5 (SD 32.7) mm^2^. The results showed a statistically significant difference depending on gender for the size of the deep insertion with an effect size of 0.707. (*p* = 0.001) The results are graphically displayed in Fig. 4.

When comparing the other parameters, all of them showed a statistically significant difference depending on gender except the maximum height of the capsular insertion. The *p* values for the t-test for independent variables, the corresponding Cohen’s *d* and the number of needed samples per group for all measurements are displayed in Table 3.

**Table 3:** Differences of all measured parameters between genders with their *p* values for the t-test for independent variables, calculated Cohen’s d and the needed sample size per group

	t-test for independent variables	Cohen’s *d*	Needed sample size per group
Deep insertion area (DI) in mm^2^	0.002	0.707	26
Superficial insertion area (SI) in mm^2^	0.001	0.751	23
Capsular insertion (CI) area in mm^2^	0.001	1.499	7
Olecranon height (OH) in mm	0.001	1.599	6
Olecranon width (OW) in mm	0.001	1.084	12
Ulnar length in cm	0.001	0.860	18
Radial length in cm	0.001	0.872	17
Maximum height CI	0.072	–	–
Maximum height DI	0.022	3.788	3
Maximum height SI	0.032	2.324	4

**Fig. 5:**
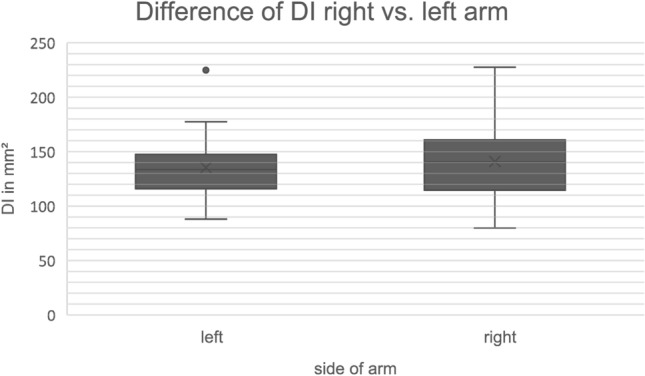
Boxplot graph visualizing the differences of the deep, muscular insertion area between right and left arm

When looking at side differences in the 41 paired specimens, we found the mean size in left specimens to be 135.2 mm^2^ and 140.9 mm^2^ in right-sided specimens. Consequently, there was no statistically significant difference in that regard (*p* = 0.401) The results are presented in Fig. 5.

Regarding correlations we found weak correlations between the ulnar length and radial length with the size of the deep muscular insertion of triceps. We also found only a weak correlation between the deep muscular insertion and the superficial tendinous insertion (Table 3).

## Discussion

In the present study we found a deep muscular insertion of the triceps and could show that it has an area of 138.1 mm^2^ and a mean anterior to posterior expansion of 5.5 mm lying in between the superficial tendinous insertion of the triceps tendon and the posterior joint capsule. Furthermore, we found the superficial tricipital insertion area to be on average 218.2 mm^2^ and the capsular insertion area 159.3 mm^2^. Additionally, we found a positive correlation between the size of the deep triceps insertion with the superficial triceps insertion, the capsular insertion, the olecranon height and width as well as ulnar and radial length. Given the mean calculated size of the deep insertion and its mean height the area could be a possible site for placement of fracture fixation devices in the future. Analyzing all measured parameters depending on the gender difference we could show statistically significant differences with medium to large effects in all of them except the maximum height of the capsular insertion (Table 4).

**Table 4:** Results calculating the Pearson Correlation coefficient r between DI as well as OH and the ulnar length, radial length, SI, CI. OH and OW. Statistically significant results are marked with*

	Ulnar length	Radial length	SI	CI	OH	OW
DI	0.314*	0.298*	0.231*	0.244*	0.250*	0.402*
OH	0.212*	0.181*	0.137*	0.028	–	0.178*

Complex fractures of the olecranon despite improvements of osteosynthetic implants remain challenging. Low-profile double-plate osteosynthesis shows a low complication rate and good clinical results [12]. Yet, soft tissue irritation and consequently the rate of hardware removal remains the main issue [13]. Compared to tension band wiring, plate osteosynthesis shows also to be more cost-efficient when including the cost for re-operations [24]. Especially small proximal olecranon fragments are difficult to stabilize with conventional plates as they are difficult to reach and on the other hand screws usually allow fixation in direction with the dislocation force factor of the triceps pull. Hence, there is still room for improvements of the available olecranon osteosynthetic implants. For this reason, the present study investigated if the dorsal surface of the olecranon—despite being the insertion for the triceps muscle and posterior capsule—could give way for the placement of osteosynthetic material. In 2006 Madsen and colleagues did an anatomic study on 8 specimens in which they described the insertional footprint of the triceps muscle on the olecranon after they exposed the tendon via a direct posterior approach and followed the tendon distally to determine whether there existed a distinct superficial and deep tendon. Additionally, three specimens were used for histologic analysis. They did not perform measurements to quantify the extent of the insertion, but concluded that the medial head has a separate insertion, lying deep to the common insertion of long and lateral head [20].

With an average of 466 mm^2^ of the insertional footprint of the triceps tendon Yeh et al. found a considerable larger area compared to our results. After dissection of the elbows to expose the triceps tendon it was dissected off its bony insertion. Subsequently, the footprint length and width was measured with a gliding digital caliper and the surface area was calculated [33]. However, their focus was on the biomechanical repair of a triceps tendon rupture and the sample size with 27 specimens was lower, compared to the present sample.

In their study of the triceps brachii muscle on the Olecranon using 100 specimens, Windisch and his colleagues, after using a posterior approach to visualize the triceps tendon, also measured the dimensions of the insertional footprint by using a digital gliding caliper. They did not differentiate between a deep and superficial insertion, only describing a common insertion on the olecranon [32].

With an olecranon width of 26.9 mm Keener et al. found a similar result compared to our 25.3 mm [18]. Further, they found a mean insertional length of 13.4 mm (range, 12.8–14.2 mm) in the proximal to distal extension and a 20.9 mm (range 19.7–22.1 mm) mean medial-to-lateral width. All measurements were done with a digital gliding caliper after sharp dissection of the triceps tendon of its insertion. Our results our considerably bigger. Yet, they concluded that there is no bipartite insertion on the olecranon. A conclusion we cannot join contemplating our findings.

In their study of the insertional footprint of the triceps and the posterior capsule Barco et al. found that the posterior capsule has an insertional footprint of 150 mm^2^ (SD 30 mm^2^) [2]. The triceps footprint was also subdivided in a superficial insertion (280 mm^2^, SD 10 mm^2^) and a deep insertion with 120 mm^2^ (SD 6 mm^2^). The capsular insertion was 159.3 mm^2^ in Barco´s study. Ten limbs were used and the proximal muscular origins were isolated and followed distally to its insertion. Laterally, the brachioradialis muscle and wrist extensors were dissected to improve distinction. After release of the capsule the deep and superficial layers of the tendon were distinguished and measured with a digital sliding caliper. The surface area was mathematically calculated. Like our findings, Barco describes a “deep muscular head of the triceps, muscular almost to the point of insertion” that is similar in its dimensions to our findings, even though our study group with 41 pair-matched elbow specimens was larger compared to the five in their study. Additionally, by measuring the surface areas digitally and not calculating them we obtain a better estimation of the real size.

That the triceps has a bipartite insertion on the olecranon was also confirmed by studies using MR-Imaging for verification. Belentani and his group studied 12 cadaveric specimens in the MRI scanner and found a bipartite insertion in all 12 specimens and correlated them with the macroscopic and microscopic anatomy. Yet, in the histological analysis they could not find a separate insertion of the three heads of the triceps muscle [3]. Negrao et al. confirmed these findings of a bipartite insertion on magnetic resonance imaging even though using a different MRI-protocol, but their histologic results could also not verify a bipartite insertion [21].

Our results of the olecranon height (OH) (26.7 mm, SD 2.3, 20.5–32.2) and the olecranon width (OW) (25.3, SD 2.4, 20.9–30.4) were similar compared to other studies [4, 7, 30].

Treatment options for olecranon fractures range from tension band wiring to intramedullary nailing to various types of plate fixation. Tension band wiring or plate fixation is the most commonly used fixation techniques [23]. Even though tension band wiring remains a suitable possibility for simple fractures with a solid proximal fragment, the techniques have their limitations when treating multifragmentary olecranon fractures, especially those with small fragments. A suitable alternative is offered through a far proximal placement of plates. Koziarz et al. found that plate osteosynthesis had a significantly lower complication rate (relative risk 0.48) and less hardware removal compared to tension band wiring [19]. Due to a higher re-operation rate and more complications with tension band wiring, plate fixation was also found to be more cost-effective [24]. Nevertheless, plate fixation with bulky implants on the other hand potentially puts soft tissue healing at risk [12]. Contemporary implants also offer medio-lateral placement at the side of the olecranon. The goal of such implants is to prevent soft tissue irritation at the dorsal olecranon as this remains one of the major complications [24]. Besides the proper placement of osteosynthetic material the proper choice of surgical approach to the elbow joint and the distal humerus, respectively, is of interest in the treatment of distal triceps avulsions. Dakouré and his group studied 30 cadaveric elbows examining the articular exposure by using a synthetic net with a mesh and compared the mean exposure of the bilatero-tricipital, triceps splitting and olecranon osteotomy approaches [8]. Olecranon osteotomy with 52% was superior in regard of exposure to the triceps splitting approach with 37% exposure and the bilatero-tricipital approach with only 26% exposure. The articular exposure as well as the risk of injury to the extensor apparatus and to the vascularization should be taken in to account. Regarding these two characteristics all commonly used approaches have their weaknesses. Therefore, a digastric olecranon osteotomy as a new approach to the elbow could pose a viable alternative, as a study of 18 elbows showed equivalent results concerning exposure, preservation of the main vascularization and preservation of the extensor apparatus, especially the triceps and anconeus muscle [6]. Additionally, better understanding of elbow anatomy, especially the course of nerve supply, will aid the post operative patients’ satisfaction. A study conducted on 54 elbows accurately displayed the course of the anconeus muscle and its main nerve supply and showed that most approaches to the elbow possibly injure the triceps anconeus nerve and subsequently lead to a atrophy of the muscle, possibly reducing its biomechanical function as an elbow stabilizer [6].

In the case of distal triceps ruptures, van Riet and his group stated that early surgical repair is the treatment of choice for distal tendon ruptures [27]. The surgical possibilities vary widely from cruciate repairs, usage of suture anchors to anatomic repair. Biomechanical analysis of 27 cadaveric elbows showed that anatomic repair better restores the insertional footprint under cyclic loading compared to other techniques [33]. However, suture anchor repair showed lower re-rupture rates in a recent systematic review including 565 triceps tendon ruptures [1]. As we could show a bipartite insertion of the triceps muscle, a double-row technique as a close anatomical reconstruction must be discussed and should be analyzed biomechanically in further studies.

The aim of this study was to define the areas of the olecranon, give a better understanding of the insertional footprint of the triceps tendon and evaluate it for possible placement of osteosynthetic material without a biomechanical relevant injury. We believe that the present study is a contribution to the available body of literature to aid in future implant developments and gives surgeons a better understanding of the tendon’s anatomy. A solution could lie in pre-shaped anatomical implants with a smaller proximal width that are placed beneath the superficial tendinous insertion despite a partial removal of the deep insertion. Further studies, especially biomechanical studies, should show that blunt dissection of the deep triceps insertion alters the biomechanical function of the triceps muscle and if the risk–benefit-profile is in favour of the patients benefit as well as biomechanical analysis of a double-row reconstruction in distal triceps avulsions.

However, there are certain limitations to this study. By using the arm holder and a standardized protocol to dissect, photograph, and measure the elbow, we tried to minimize errors as far as possible, yet measuring mistakes can still occur and can influence the results. Furthermore, we performed the study on embalmed specimens. While recent literature describes a shrinkage of bone and muscle tissue, the insertions of capsules and muscles should not be affected. Likewise, bony structures should not be affected as the soft tissue shrinks due to fixation [5]. The mean specimen age was 83.9 years, which rather corresponds to the typical total elbow replacement cohort age than the main age group for olecranon fractures. Because we excluded all specimens with previous procedures to the upper extremity or degenerative changes to the elbow, we believe that the high age does not alter the anatomical relations and structures in a relevant extend.

## Conclusion

On the posterior aspect of the olecranon tip we can find a bipartite insertion of the triceps muscle, with a deep, muscular part and a superficial, tendinous part. The deep part would be a possible site for the placement of new types of plates in the osteosynthesis of fractures in order to minimize plate exposure and irritation the

 main part of the triceps tendon. The size of the deep insertion area of the triceps correlates to the length of ulna and radius, the size of the capsular and superficial tendinous insertion as well as the olecranon height and width. In males the size of the area is statistically significantly larger than in females. Reconstruction of the distal triceps in avulsions could be performed in a double-row technique to further facilitate postoperative function.

## Supplementary Information

Below is the link to the electronic supplementary material.

Supplementary file1 (PDF 793 KB)

Supplementary file2 (PDF 1552 KB)
