# Supplementary material for: Anatomic evaluation of the triceps tendon insertion at the proximal olecranon regarding placement of fracture fixation devices
Source: Surg Radiol Anat. 2022 Mar 17;44(4):627–34. doi: 10.1007/s00276-022-02921-y (PMC8960582; doi:10.1007/s00276-022-02921-y)
Supplement: Supplementary file 1 — Supplementary file1 (PDF 793 KB) [file 276_2022_2921_MOESM1_ESM.pdf]

Universität zu Köln

Geschäftsstelle Ethikkommission • Universität zu Köln • 50931 Köln

Uniklinik Köln, Klinik und Poliklinik für
Orthopädie und Unfallchirurgie
Sebastian Wegmann
Kerpener Str. 62
50937 Köln

Per Fax:
0221 478 4835

Köln, 22.01.2020

Unser Zeichen: 19-1632

Anatomisch deskriptive Studie zur Insertion von Muskel-/Bandstrukturen am Olekranon

Sehr geehrter Herr Dr. Wegmann,

die Ethikkommission der Medizinischen Fakultät der Universität zu Köln hat sich in ihrer Sitzung vom 16.01.2020 mit Ihrem Antrag befasst. Die Beratung erfolgte nach § 15 Abs. 1 der Berufsordnung der Nordrheinischen Ärztinnen und Ärzte in Verbindung mit § 2 Abs. 1 der Satzung für die Ethikkommission der Medizinischen Fakultät der Universität zu Köln.

Der Antrag wird **zustimmend bewertet**.

Begründung

Die Unterlagen, einschließlich des Studienplans und der Modalitäten entsprechen dem Stand der wissenschaftlichen Erkenntnisse. Die vorhersehbaren Risiken und Nachteile der Studie sind gegenüber dem Nutzen und der voraussichtlichen Bedeutung der Ergebnisse für die Heilkunde ärztlich vertretbar.

Hinweise

Wir dürfen Sie darum bitten, die Ethikkommission unverzüglich von sämtlichen nachträglichen Änderungen im Studienplan (abgesehen von rein formellen) zu unterrichten, da sie eine erneute Beratung erforderlich machen.

Die Ethikkommission bittet um Unterrichtung über den Beginn der Studie sowie ferner über einen möglichen frühzeitigen Abbruch der Studie. Wir bitten um Übersendung eines jährlichen Zwischenberichtes Nach Abschluss des Projektes bitten wir um Übersendung eines Schlussberichtes.

Medizinische Fakultät der Universität zu Köln

Geschäftsstelle der Ethikkommission

Vorsitzender
Univ.-Prof. Dr. med.
Raymond Voltz

Leitung der Geschäftsstelle
Dr. med. Guido Grass
Telefon +49 221 478 87916

Stellv. Leitung
Dipl.-Ges.-Ök. Karolina Mäder
Telefon +49 221 478 88844

Dipl.-Biol. Alice Follmann
Telefon +49 221 478 97772

Dipl.-Ges.-Ök. Christine Grimm
Telefon +49 221 478 87488

Dipl.-Ges.-Ök. Agnieszka Hompanera Torre
Telefon +49 221 478 82903

Corinna Wendeler M.Sc.
Telefon +49 221 478 82902

Christin Willgrod M.A.
Telefon +49 221 478 97773

Büroleitung
Barbara Ulhardt M.A.
Telefon +49 221 478 82900
Telefax +49 221 478 82905

ek-med@uni-koeln.de
www.ek-koeln.de

Servicezeiten:
Mo. – Do. 9.00 – 16.00 Uhr
Fr. 9.00 – 12.00 Uhr
und nach Vereinbarung

Besucheradresse:
Gleueler Str. 269
50937 Köln

Postanschrift:
Kerpener Str. 62
50937 Köln

Bankverbindung:
Bank für Sozialwirtschaft Köln
BLZ 370 205 00
Kto.-Nr. 8 150 000
BIC BFSWDE31
