# Supplementary material for: Anatomic evaluation of the triceps tendon insertion at the proximal olecranon regarding placement of fracture fixation devices
Source: Surg Radiol Anat. 2022 Mar 17;44(4):627–34. doi: 10.1007/s00276-022-02921-y (PMC8960582; doi:10.1007/s00276-022-02921-y)
Supplement: Supplementary file 2 — Supplementary file2 (PDF 1552 KB) [file 276_2022_2921_MOESM2_ESM.pdf]

Schreiben der Ethikkommission vom 22.01.2020
Antragsnummer 19-1632

Die Ethikkommission der Medizinischen Fakultät der Universität zu Köln setzt sich zusammen und arbeitet gemäß den nationalen gesetzlichen Bestimmungen. Hierbei werden die Grundsätze, wie sie in der „Note for Guidance on Good Clinical Practice“ (CPMP/ICH/135/95) niedergelegt sind, berücksichtigt.

Entsprechend der Funktion der Ethikkommission betrifft diese Stellungnahme nur die ethische Beurteilung der Konzeption, der vorgesehenen Methoden, Durchführung und Überwachung des betreffenden Projektes sowie der beabsichtigten Patientenaufklärung. Die ärztliche und juristische Verantwortung verbleibt jedoch uneingeschränkt beim Projektleiter und seinen Mitarbeitern, so dass alle zivil- oder haftungsrechtlichen Folgen, die sich ergeben könnten, von dieser Seite zu tragen sind.

Mit freundlichen Grüßen

Prof. Dr. Raymond Voltz

Dipl.-Ges.-Ök. Karolina Mäder

Liste der Beschluss fassenden Kommissionsmitglieder

Dr. Anna-Maria Fink

Prof. Dr. Ioanna Gouni-Berthold

Dr. Anne Koy

Dr. Vanessa Romotzky

Prof. Dr. Raymond Voltz (Vorsitz)

Folgende Unterlagen haben zur Beratung vorgelegen

1) Anschreiben Ethikkommission.pdf vom 16.12.2019
2) Antrag Ethikkomission.pdf vom 16.12.2019

Das Votum ist gültig für folgende Studienzentren

Prof. Dr. Lars Peter Müller,
PD Dr. Kilian Wegmann,
Dr. Sebastian Wegmann,
Uniklinik Köln, Klinik und Poliklinik für Orthopädie und Unfallchirurgie
Kerpener Str. 62
50937 Köln
